# Comparison of vestibular function in hereditary hearing loss patients with *GJB2*, *CDH23,* and *SLC26A4* variants

**DOI:** 10.1038/s41598-024-61442-3

**Published:** 2024-05-08

**Authors:** Keita Tsukada, Shin-ya Nishio, Yutaka Takumi, Shin-ichi Usami

**Affiliations:** 1 Department of Otorhinolaryngology Head and Neck Surgery, Shinshu University School of Medicine, 3-1-1 Asahi, Matsumoto, 390-8621 Japan; 2 Department of Hearing Implant Sciences, Shinshu University School of Medicine, 3-1-1 Asahi, Matsumoto, 390-8621 Japan

**Keywords:** Genetics, Neurological disorders

## Abstract

To investigate the association between hereditary hearing loss and vestibular function, we compared vestibular function and symptoms among patients with *GJB2*, *SLC26A*4, and *CDH23* variants. Thirty-nine patients with sensory neural hearing loss (11 males and 28 females) with biallelic pathogenic variants in either *GJB2*, *SLC26A4*, or *CDH23* were included in this study (13 *GJB2*, 15 *SLC26A4*, and 11 *CDH23*). The patients were examined using caloric testing and cervical and ocular vestibular-evoked myogenic potentials (cVEMP and oVEMP). We also compared vestibular function and symptoms between patients with these gene variants and 78 normal-hearing ears without vestibular symptoms as controls. The frequency of semicircular canal hypofunction in caloric testing was higher in patients with *SLC26A4* variants (47%) than in those with *GJB2* (0%) and *CDH23* variants (27%). According to the cVEMP results, 69% of patients with *GJB2* variants had saccular hypofunction, a significantly higher proportion than in those carrying other variants (*SLC26A4*, 20%; *CDH23*, 18%). In oVEMP, which reflects utricular function, no difference was observed in the frequency of hypofunction among the three genes (*GJB2*, 15%; *SLC26A4*, 40%; and *CDH23*, 36%). Hence, discernable trends indicate vestibular dysfunction associated with each gene.

## Introduction

Congenital hearing loss (HL) occurs in 1 in 700–1000 newborns, with an estimated 50–70% of cases attributed to genetic causes^1^. Over 120 genes have been identified as causative factors for non-syndromic HL^2^.

Our previous large cohort analysis using massive parallel DNA sequencing showed that *GJB2* variants were the most frequent cause of HL, followed by *SLC26A4* (MIM #605,646) and *CDH23* (NM_22124) variants. Pathogenic variants in these three genes comprise 32% of the genes responsible for pre-lingual onset HL^3,4^.

*GJB2* encodes the Connexin 26 (Cx26) protein, a member of the Connexin family, and functions as a gap junction protein that maintains cochlear homeostasis and the circulation of metabolites and is important for cellular function and survival of supporting cells^5,6^. More than 450 *GJB2* variants have been reported as causative factors for HL (HGMD professional 2023.4, QIAGEN, Hilden, Germany). Hearing levels range from mild to profound according to different genotypes, and HL is thought to be non-progressive^7^.

Variants in the *CDH23* gene are known to be the cause of both Usher syndrome type ID (USH1D) and non-syndromic HL (DFNB12)^8,9^. This gene encodes Cadherin 23, an important component of the tip link that maintains the arrangement of stereocilia^10,11^. More than 650 variants have been reported for USH1D and DFNB12 phenotypes (HGMD professional 2023.4)^8,9,12–14^. As suggested by a genotype–phenotype correlation study, *CDH23* null alleles cause USH1D, characterized by congenital profound HL, vestibular dysfunction, and late-onset retinitis pigmentosa, whereas most missense variations cause non-syndromic HL (DFNB12)^8,9,12–15^, with a wide phenotype spectrum ranging from congenital severe-to-profound HL to late-onset high-frequency HL^16^.

Pathogenic variants in the *SLC26A4* gene are responsible for a broad phenotypic spectrum, ranging from typical Pendred syndrome to non-syndromic HL (DFNB4)^17^. Most patients with *SLC26A4* variants have inner ear malformations, with the most frequent feature being an enlarged vestibular aqueduct (EVA)^18,19^. The typical clinical characteristics of patients with EVA caused by biallelic *SLC26A4* variants include fluctuating and progressive HL, often associated with vertigo and/or goiter^17,20^.

These genes are also expressed in peripheral vestibular organs, suggesting the presence of similar sensory functions^21^. Therefore, patients with variants in these major causative genes of HL may exhibit a phenotype of vestibular dysfunction in addition to the phenotype of HL.

Although numerous reports have addressed HL, few have focused on or compared the features of vestibular symptoms and/or functions resulting from these three gene variants. In this study, we compared vestibular symptoms and functions among patients with HL caused by *GJB2* and *SLC26A4* variants with those having DFNB12 caused by *CDH23* variants. We aimed to investigate the presence of vestibular symptoms and dysfunction in patients with variants in these major genes and identify potential differences among them.

## Methods

### Patients

This observational comparative study included 39 patients with sensory-neural HL (11 males and 28 females) with biallelic pathogenic variants in either *GJB2*, *SLC26A4*, or *CDH23* after obtaining informed written consent. The age at vestibular testing ranged from 8 to 54 years, and the mean age was 24.4 ± 15.8 years. None of the patients had a history of cochlear implant surgery. The data regarding the age and sex of patients associated with each gene related to HL are shown in Table 1. Thirteen patients (2 males and 11 females) with *GJB2* variants, 15 (5 males and 10 females) with *SLC26A4* variants, and 11 (4 males and 7 females) with *CDH23* variants underwent vestibular testing (caloric, cVEMP, and oVEMP). No statistically significant differences were observed in age or sex among patients with gene variants. Although no inner ear anomalies were observed in patients with *GJB2* and *CDH23* variants, all patients with *SLC26A4* variants had bilateral EVA, diagnosed using a computed tomography scan (according to the criteria of EVA: a diameter > 1.5 mm at the midpoint between the common crus and external aperture).

**Table 1:** Summary of age, gene variants, and hearing levels in patients with each gene.

	*GJB2*	*SLC26A4*	*CDH23*	*P*-value
	(n = 13)	(n = 15)	(n = 11)	
Age, mean ± SD	23.7 ± 15.4	21.0 ± 14.8	29.6 ± 17.4	0.30
Sex, n (%)				
Male	2 (15)	5 (33)	4 (36)	0.45
Female	11 (85)	10 (67)	7 (64)	
Hearing levels (dB), mean ± SD	78.3 ± 27.3	84.3 ± 19.6	94.2 ± 7.5	0.86
Mild, ear (%)	2 (8)	0 (0)	0 (0)	
Moderate, ear (%)	8 (31)	7 (23)	0 (0)	
Severe, ear (%)	5 (19)	14 (47)	5 (23)	
Profound, ear (%)	11 (42)	9 (30)	17 (73)	

SD, standard deviation; dB, decibel.

For the controls, vestibular testing was performed on 39 healthy participants with normal hearing and devoid of vestibular symptoms, as well as 175 unaffected ears with normal hearing in patients with unilateral HL resulting from mumps, unilateral cochlear nerve deficiency, or sudden deafness. Among the 214 healthy controls, 161 underwent caloric testing, and 173 and 121 underwent cVEMP and oVEMP, respectively. We selected 78 ears (22 male and 56 female) for each vestibular test as age- and sex-matched controls. The mean age was 24.6 ± 16.2 (range 7–58), 24.7 ± 16.5 (range 8–60), and 23.6 ± 16.4 (range 8–58) years in individuals who underwent caloric testing, cVEMP, and oVEMP, respectively. This study was approved by the respective ethical committees of the Shinshu University Ethical Committee (approval number: 718) and was conducted in accordance with the Declaration of Helsinki.

### Genetic analysis

To confirm the presence of *GJB2*, *SLC26A4*, and *CDH23* variants, a DNA fragment containing all the exons of each gene, including flanking intronic sequences, was sequenced using a massively parallel DNA sequencer. Any potential pathological variants were selected and confirmed following procedures described elsewhere^15,17,22^. All patients exhibited biallelic variants in each gene. All patients with *CDH23* variants had biallelic missense variants, implying that they had DFNB12 and not USH1D.

### Audiological evaluation

Hearing levels were determined using pure-tone audiometry (PTA). The average threshold in the conversation frequencies (0.5 kHz, 1.0 kHz, 2.0 kHz, and 4.0 kHz) was calculated, and the severity of hearing loss in the better hearing ear was categorized as mild (20–39 dB), moderate (40–69 dB), severe (70–89 dB), or profound (≥ 90 dB). A summary of the hearing levels associated with each gene is shown in Table 1.

### Vestibular testing

The patients were examined using caloric testing, cVEMP, and oVEMP to obtain data on semicircular canal and otolithic functions (saccular and utricular functions, respectively).

For cVEMP testing, electromyography (EMG) was performed using a pair of surface electrodes mounted on the upper half and sternal head of the sternocleidomastoid (SCM) muscle. Electrographic signals were recorded using a Neuropack-evoked potential recorder (Nihon Kohden Co., Ltd., Tokyo, Japan). Clicks lasting for 0.1 ms at 105 dBnHL were delivered via headphones. The stimulation rate was 5 Hz, with a bandpass filter intensity of 20–2000 Hz, and an analysis time of 50 ms. Responses to 200 stimuli were averaged twice. Because the amplitude of the cVEMP, based on the unrectified EMG, correlates with SCM muscle activity during the test^23^, we measured SCM muscle activity using the background integrated EMG response, referred to as the area under the averaged rectified EMG curve, from − 20 to 0 ms before sound stimulation. In cVEMP, the amplitude was defined as the difference between peaks p13 and n23. Correction between p13 and n23 amplitudes was calculated as follows^24^:

$$Corrected\;amplitude = amplitude\;of\;the\;averaged\;unrectified\;EMG\;\left( {microV} \right)/background\;integrated\;EMG\;\left( {microV} \right).$$

oVEMP testing was performed using bone conductive vibration (BCV). BCV comprised 4 ms tone bursts of 500 Hz vibration (rise/fall time = 1 ms and plateau time = 2 ms), administered via a handheld 4810 mini-shaker (Bruel and Kjaer, Naerum, Denmark), positioned on the forehead at the midline (Fz). The active electrode was situated over the inferior orbital margin, whereas a reference electrode was placed 2 cm below the active electrode. The ground electrode was then placed on the chin. The patients lay in a supine position on the bed and looked approximately 30° above straight ahead during the recording. The signals were amplified and bandpass-filtered between 20 and 2000 Hz. The stimulus intensity was set at 115 dB force level, with a frequency of 500 Hz, an analysis time of 40 ms, and 50 responses were averaged for each run. For oVEMP, the amplitude was defined as the difference between peaks n10 and p15.

In caloric testing, maximum slow phase velocity (MSPV) was measured using cold water irrigation (20 °C, 5 mL, 20 s). We defined MSPV below 10°/s as areflexia and between 10° and 20°/s as hyporeflexia.

### Statistical analysis

Statistical Package for the Social Sciences version 26 for Windows (IBM Co., Chicago, IL, USA) was used for all analyses, and the Kruskal–Wallis test was used to compare differences in age and PTA among the three groups. Mann–Whitney U tests were employed to compare differences in vestibular testing between patients with each gene variant and normal controls. The correlation between PTA severity, age, and vestibular function was calculated using Spearman’s correlation coefficient. Fisher’s exact test was utilized to compare the frequencies of vestibular symptoms, semicircular canals, and saccular and utricular hypofunction between patients with each gene variant. Statistical significance was set at *P* < 0.05.

## Results

Details of the gene variants and vestibular and audiological test results for all patients are shown in Table 2 and Supplementary Table S1.

**Table 2:** Summary of gene variants and vestibular and audiological results for all patients.

No	Gene	Age	Sex	Base change	AA change	Pathogenicity	Vestibular symptoms	PTA (R) dB	PTA (L) dB	Hearing levels	Caloric testing (R)	Caloric testing (L)	cVEMP (R)	cVEMP (L)	oVEMP (R)	oVEMP (L)
1	*GJB2*	18	F	c.[235*del*C];[279G > A]	p.[L79Cfs*3];[M93I]	Pathogenic, Likely pathogenic	−	57	56	Moderate	+	+	Decreased	+	+	+
2	*GJB2*	19	F	c.[176–191 *del*16];[235*del*C]	p.[G59Afs*18];[L79Cfs*3]	Pathogenic, Pathogenic	+	105	105	Profound	+	+	+	+	+	+
3	*GJB2*	17	M	c.[109G > A];[109C > A]	p.[V37I];[V37I]	Pathogenic, Pathogenic	−	48.8	43.8	Moderate	+	+	+	+	+	+
4	*GJB2*	9	F	c.[134G > A; 408C > A];[299-300*de*lAT]	p.[G45E;Y136X];[H100Rfs*14]	Pathogenic, Pathogenic	−	76.7	68.3	Severe	+	+	+	+	+	+
5	*GJB2*	20	F	c.[235*del*C];[235*del*C]	p.[L79Cfs*3];[L79Cfs*3]	Pathogenic, Pathogenic	−	105	103.3	Profound	+	+	Absent	+	+	+
6	*GJB2*	16	F	c.[134G > A; 408C > A];[235delC]	p.[G45E;Y136X];[L79Cfs*3]	Pathogenic, Pathogenic	−	55	46.3	Moderate	+	+	Decreased	Absent	+	+
7	*GJB2*	11	F	c.[134G > A; 408C > A];[235delC]	p.[G45E;Y136X];[L79Cfs*3]	Pathogenic, Pathogenic	−	86.3	87.5	Severe	+	+	Decreased	+	+	+
8	*GJB2*	9	F	c.[134G > A; 408C > A];[235delC]	p.[G45E;Y136X];[L79Cfs*3]	Pathogenic, Pathogenic	−	103.8	83.8	Severe	+	+	+	+	+	+
9	*GJB2*	41	F	c.[235*del*C];[299-300*del*AT]	p.[L79Cfs*3];[H100Rfs*14]	Pathogenic, Pathogenic	−	108.8	105	Profound	+	+	Decreased	+	Decreased	Decreased
10	*GJB2*	38	F	c.[235*del*C];[235*del*C]	p.[L79Cfs*3];[L79Cfs*3]	Pathogenic, Pathogenic	−	56.3	56.4	Moderate	+	+	+	Absent	+	+
11	*GJB2*	11	M	c.[109G > A];[109C > A]	p.[V37I];[V37I]	Pathogenic, Pathogenic	−	30	30	Mild	+	+	Decreased	Decreased	+	+
12	*GJB2*	54	F	c.[134G > A; 408C > A];[235delC]	p.[G45E;Y136X];[L79Cfs*3]	Pathogenic, Pathogenic	−	105	105	Profound	+	+	Absent	Absent	+	Decreased
13	*GJB2*	46	F	c.[134G > A; 408C > A];[235delC]	p.[G45E;Y136X];[L79Cfs*3]	Pathogenic, Pathogenic	−	105	105	Profound	+	+	Absent	Absent	+	+
14	*SLC26A4*	15	F	c.[2168A > G];[2168A > G]	p.[H723R];[H723R]	Pathogenic, Pathogenic	+	77.5	87.5	Severe	Hyporeflexia	Hyporeflexia	+	+	+	+
15	*SLC26A4*	17	M	c.[1651dup];[2168A > G]	p.[S551Ffs*13];[H723R]	Pathogenic, Pathogenic	+	60	62.5	Moderate	Hyporeflexia	+	+	+	+	+
16	*SLC26A4*	27	F	c.[2168A > G];[2168A > G]	p.[H723R];[H723R]	Pathogenic, Pathogenic	+	78.8	100	Severe	Areflexia	+	+	+	+	+
17	*SLC26A4*	18	M	c.[919-2A > G];[2168A > G]	p.[spl.];[H723R]	Pathogenic, Pathogenic	+	85	53.8	Moderate	+	hyporeflexia	+	+	+	+
18	*SLC26A4*	8	F	c.[1229C > T];[1229C > T]	p.[T410M];[T410M]	Pathogenic, Pathogenic	+	80	82	Severe	+	+	+	+	Absent	Absent
19	*SLC26A4*	16	F	c.[1229C > T];[1229C > T]	p.[T410M];[T410M]	Pathogenic, Pathogenic	+	88.8	91.3	Severe	+	+	+	+	Decreased	Decreased
20	*SLC26A4*	9	F	c.[1174A > T];[1707 + 5G > A]	p.[N392Y];[spl.]	Pathogenic, Pathogenic	−	80	102	Severe	+	+	+	+	+	+
21	*SLC26A4*	13	M	c.[919-2A > G];[1229C > T]	p.[spl.];[T410M]	Pathogenic, Pathogenic	+	85	88.8	Severe	+	+	+	+	+	+
22	*SLC26A4*	48	F	c.[2168A > G];[2168A > G]	p.[H723R];[H723R]	Pathogenic, Pathogenic	+	78.8	90	Severe	Areflexia	Areflexia	Absent	Absent	Absent	Absent
23	*SLC26A4*	41	F	c.[919-2A > G];[2168A > G]	p.[spl.];[H723R]	Pathogenic, Pathogenic	+	105	87.5	Severe	+	+	Decreased	+	Decreased	+
24	*SLC26A4*	7	M	c.[1174A > T];[2168A > G]	p.[N392Y];[H723R]	Pathogenic, Pathogenic	−	58.8	62.5	Moderate	+	+	+	+	+	+
25	*SLC26A4*	9	F	c.[919-2A > G];[2168A > G]	p.[spl.];[H723R]	Pathogenic, Pathogenic	+	87.5	65	Moderate	Hyporeflexia	Areflexia	+	+	+	Decreased
26	*SLC26A4*	8	M	c.[2168A > G];[2168A > G]	p.[H723R];[H723R]	Pathogenic, Pathogenic	−	66.3	62.5	Moderate	+	+	+	+	+	+
27	*SLC26A4*	51	F	c.[2168A > G];[2168A > G]	p.[H723R];[H723R]	Pathogenic, Pathogenic	+	103.8	96.3	profound	Hyporeflexia	Areflexia	Decreased	+	Decreased	Decreased
28	*SLC26A4*	28	F	c.[2168A > G];[2168A > G]	p.[H723R];[H723R]	Pathogenic, Pathogenic	−	105	100	Profound	+	+	+	+	+	+
29	*CDH23*	16	M	c.[719C > T];[5147A > C]	p.[P240L];[Q1716P]	Pathogenic, Pathogenic	−	97	99	Profound	+	+	+	+	+	+
30	*CDH23*	52	F	c.[719C > T];[4249C > T]	p.[P240L];[R1417W]	Pathogenic, VUS	+	101.3	70	Severe	+	+	Decreased	+	Decreased	Decreased
31	*CDH23*	51	F	c.[4249C > T];[4249C > T]	p.[R1417W];[R1417W]	VUS, VUS	−	97.5	100	Profound	Hyporeflexia	+	+	+	+	+
32	*CDH23*	53	M	c.[4249C > T];[4249C > T]	p.[R1417W];[R1417W]	VUS, VUS	−	98.8	92.5	Profound	+	Hyporeflexia	Decreased	+	Absent	Absent
33	*CDH23*	46	M	c.[719C > T];[4762C > T]	p.[P240L];[R1588W]	Pathogenic, Pathogenic	−	101.3	101	Profound	+	+	+	+	+	Decreased
34	*CDH23*	21	F	c.[719C > T];[4762C > T]	p.[P240L];[R1588W]	Pathogenic, Pathogenic	−	97.5	95	Profound	+	+	+	+	+	Decreased
35	*CDH23*	13	F	c.[719C > T];[2866G > A]	p.[P240L];[E956K]	Pathogenic, Pathogenic	−	88.8	91.3	Severe	+	+	+	+	+	+
36	*CDH23*	23	F	c.[4249C > T];[4249C > T]	p.[R1417W];[R1417W]	VUS, VUS	−	97.5	96.3	Profound	+	+	+	+	+	+
37	*CDH23*	8	F	c.[719C > T];[7312G > A ]	p.[P240L];[E2438K]	Pathogenic, Pathogenic	−	83.8	92.5	Severe	+	+	+	+	+	+
38	*CDH23*	28	F	c.[719C > T]:[6085C > T]	p.[P240L];[R2029W]	Pathogenic, Pathogenic	−	100	98.8	Profound	Hyporeflexia	Hyporeflexia	+	+	+	+
39	*CDH23*	15	M	c.[719C > T];[902G > A]	p.[P240L];[R301Q]	Pathogenic, Pathogenic	−	88	85	Severe	+	+	+	+	+	+

All variants are indicated on NM_004004 for GJB2, NM_000441 for SLC26A4, and NM_022124 for CDH23.

cVEMP, cervical vestibular-evoked myogenic potentials; oVEMP, ocular vestibular-evoked myogenic potentials; PTA, pure-tone audiometry. Caloric testing, cVEMP, oVEMP; + normal.

### Vestibular function

Nine out of 13 patients (69%) with *GJB2* variants, 11 out of 15 patients (73%) with *SLC26A4* variants, and 6 out of 11 patients (55%) with *CDH23* variants exhibited at least one form of vestibular dysfunction, either caloric testing, cVEMP, or oVEMP.

### Semicircular canal function

**Figure 1:**
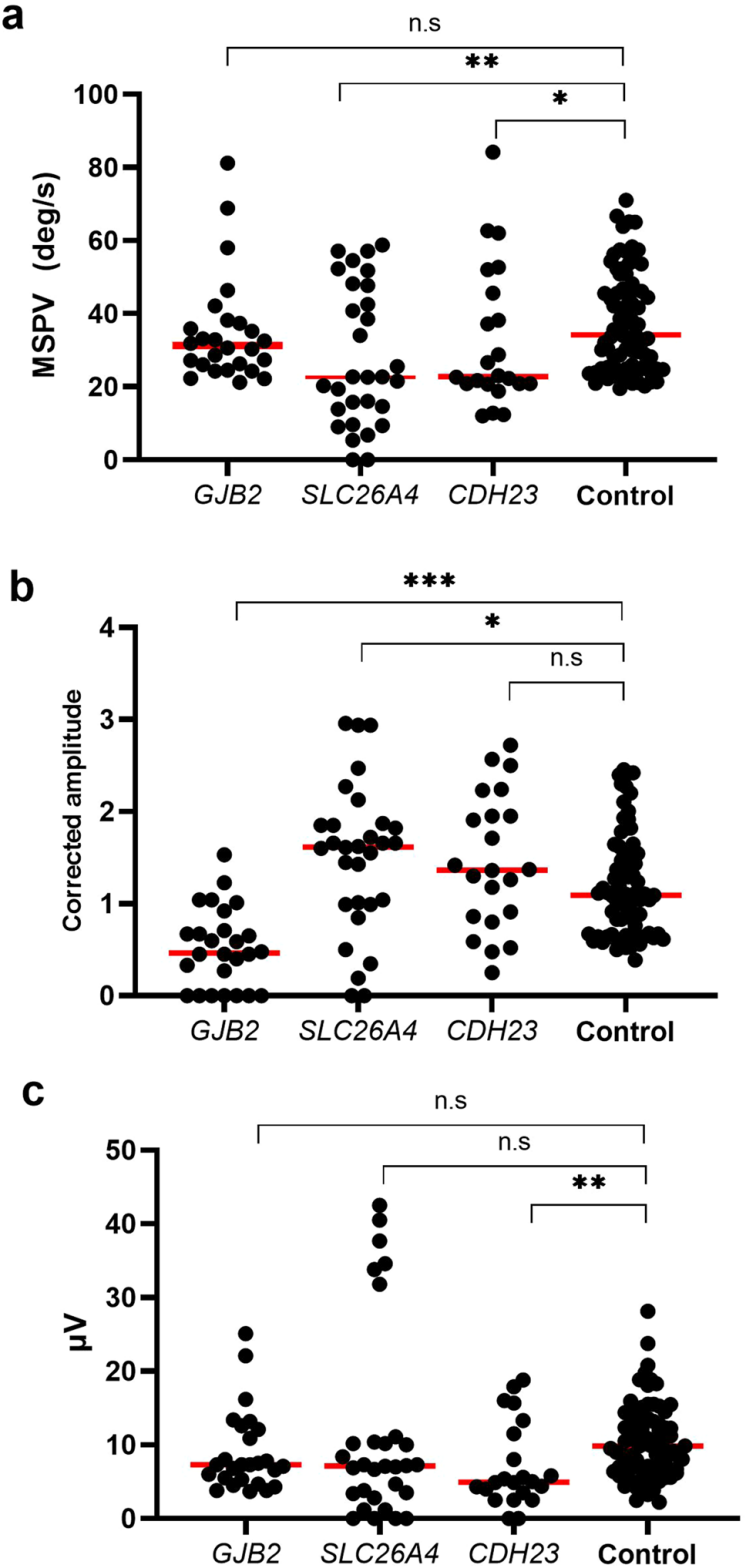
Comparisons between vestibular tests for the ears with *GJB2*, *SLC26A4,* and *CDH23* variants and normal controls. (**a**) A comparison between MSPV of caloric testing for the ears with *GJB2*, *SLC26A4,* and *CDH23* variants and normal controls. The MSPV in patients with *SLC26A4* and *CDH23* variants was significantly lower than that in controls (*P* = 0.005 in *SLC26A4* and *P* = 0.023 in *CDH23*). (**b**) A comparison between corrected amplitudes of cVEMP for the ears with *GJB2*, *SLC26A4,* and *CDH23* variants and controls. The corrected amplitude of cVEMP in patients with *GJB2* variants was statistically lower, and that in patients with *SLC26A4* variants was higher than that in controls (*P* < 0.001 for *GJB2* and *P* = 0.027 for *SLC26A4*). (**c**) A comparison between amplitudes of oVEMP for the ears with *GJB2*, *SLC26A4,* and *CDH23* variants and normal controls. A significantly lower amplitude in patients with *CDH23* variants was shown compared with that in normal controls (*P* = 0.0028). cVEMP, cervical vestibular-evoked myogenic potentials; oVEMP, ocular vestibular-evoked myogenic potentials; MSPV, maximum slow phase velocity.

Figure 1a shows a comparison between the MSPV of caloric testing for ears with *GJB2*, *SLC26A4*, and *CDH23* variants and normal controls. The median MSPV was 31.3°/s in ears with *GJB2* variants, 22.6°/s in ears with *SLC26A4* variants, 22.8°/s in ears with *CDH23* variants, and 34.2°/s in normal controls. Although no significant difference was observed in MSPV between patients with *GJB2* variants and controls (*P* = 0.39), the MSPV in patients with *SLC26A4* and *CDH23* variants was significantly lower than that in controls (*P* = 0.005 for *SLC26A4* and *P* = 0.023 for *CDH23*).

Regarding the frequency of abnormal semicircular canal function, no patient with *GJB2* variants had pathological results in caloric testing (Table 3). Among the 15 patients with *SLC26A4* variants, eight (47%) exhibited semicircular canal dysfunction (one bilateral areflexia, two unilateral areflexia or hyporeflexia in each ear, one bilateral hyporeflexia, one unilateral areflexia, and one unilateral hyporeflexia). Among the patients with *CDH23* variants, three out of 11 (27%) had semicircular canal dysfunction (one bilateral hyporeflexia and two unilateral hyporeflexia). Patients with *GJB2* variants displayed a significantly lower incidence of semicircular canal dysfunction than those with variants in the other two genes (*P* = 0.012). Although the frequency of semicircular canal dysfunction in patients with *SLC26A4* variants was higher than that in those with *CDH23* variants, the difference was not statistically significant (*P* = 0.428).

**Table 3:** Frequencies of vestibular function in each vestibular testing and symptoms.

	*GJB2*	*SLC26A4*	*CDH23*	*P*-value
	(n = 13)	(n = 15)	(n = 11)	
Vestibular dysfunction, n (%)				
Normal	4 (31)	4 (27)	5 (45)	
Pathological	9 (69)	11 (73)	6 (55)	
Caloric testing, n (%)				0.012
Normal	13 (100)	8 (53)	8 (73)	
Pathological	0	7 (47)	3 (27)	
cVEMP, n (%)				0.014
Normal	4 (31)	12 (80)	9 (82)	
Pathological	9 (69)	3 (20)	2 (18)	
oVEMP, n (%)				0.38
Normal	11 (85)	9 (60)	7 (64)	
Pathological	2 (15)	6(40)	4 (36)	
Vestibular symptoms, n (%)				< 0.001
Yes	1 (8)	11 (73)	1 (9)	
No	12 (92)	4 (27)	10 (91)	

cVEMP, cervical vestibular-evoked myogenic potentials; oVEMP, ocular vestibular-evoked myogenic potentials.

### Saccular function

Figure 1b shows the comparison between the corrected amplitudes of cVEMP in patients with *GJB2*, *SLC26A4, or CDH23* variants and controls. The median corrected amplitudes were 0.47, 1.61, 1.37, and 1.09 in patients with *GJB2*, *SLC26A4*, *CDH23* variants, and controls, respectively. The corrected amplitude of cVEMP in patients with *GJB2* variants was statistically lower, and that in patients with *SLC26A4* variants was higher than that in normal controls (*P* < 0.001 for *GJB2* and *P* = 0.027 for *SLC26A4*).

Fourteen out of all patients (35.9%) and 19 out of 78 ears (24.4%) showed no reaction or a value lower than 0.52, which was the cut-off for amplitude in the cVEMP results for the lowest 5% of controls. Nine out of the 13 patients (69%) with *GJB2* variants had pathological results of cVEMP (five patients unilaterally and four patients bilaterally). Among patients with *SLC26A4* and *CDH23* variants, three out of 15 (20%) and two out of 11 (18%) patients exhibited decreased or absent cVEMP reactions, respectively (Table 3). The frequency of saccular hypofunction was significantly higher in patients with *GJB2* variants than in those with *SLC26A4* and *CDH23* variants (*P* = 0.014).

### Utricular function

In oVEMP, the median n10-p15 amplitudes in patients with *GJB2*, *SLC26A4*, and *CDH23* variants were 7.3, 7.1, and 4.9 μV, respectively.

Comparing the n10-p15 amplitude of oVEMP between patients with variants in each gene and normal controls (Fig. 1c), significantly lower amplitudes were observed in patients with *CDH23* variants (*P* = 0.0028) than in normal controls (median 9.9 μV).

Thirteen out of all patients (33.3%) and 21 out of 78 ears (26.9%) exhibited no reaction or a value lower than 3.9 μV, which was the cut-off for amplitude in the oVEMP results for the lowest 5% of normal controls. The frequencies of utricular hypofunction were 2/13 (15%) in patients with *GJB2* variants, 6/15 (40%) in those with *SLC26A4* variants, and 4/11 (36%) in those with *CDH23* variants (Table 3), showing no significant difference (*P* = 0.38).

### Vestibular symptoms

Thirteen out of 39 patients (33%) complained of vestibular symptoms. Eleven out of 15 patients (73%) with *SLC26A4* variants complained of vestibular symptoms. Among patients with *SLC26A4* variants, out of the 11 patients with vestibular symptoms, eight patients (72.7%) complained of episodic vertigo, one patient (9.1%) complained of episodic vertigo and chronic dizziness, and two patients (18.2%) complained of occasional dizziness. Furthermore, 10 out of 11 patients (90.9%) with vestibular symptoms exhibited at least one form of vestibular dysfunction, as evidenced by either caloric testing (7/11, 63.6%), cVEMP (3/11, 27.2%), or oVEMP (6/11, 54.5%). However, all patients without vestibular symptoms showed normal vestibular function in caloric testing, cVEMP, and oVEMP. Only one out of 13 patients (7.7%) with *GJB2* variants and one out of 11 (9.1%) patients with *CDH23* variants complained of vestibular symptoms. Each patient with *GJB2* or *CDH23* variants complained of dizziness and had no history of vertigo. Patient No. 2 with *GJB2* variants, who complained of dizziness, had no vestibular dysfunction, whereas patient No. 30 with *CDH23* variants, who also complained of dizziness, showed decreased reactions in both cVEMP and oVEMP. The frequency of vestibular symptoms was significantly higher in patients with *SLC26A4* variants than in those with *GJB2* and *CDH23* variants (*P* < 0.001) (Table 3).

### Relationship between PTA, age, and vestibular functions

Regarding the correlation between PTA and vestibular function, no statistically significant correlations were found between PTA and MSPV, corrected amplitude of cVEMP, and amplitude of oVEMP in patients with any gene variants (Table 4).

**Table 4:** Correlation between hearing levels and vestibular function.

PTA	Caloric testing		cVEMP		oVEMP	
	Rho	*P*-value	Rho	*P*-value	Rho	*P*-value
*GJB2*	− 0.22	0.29	− 0.23	0.25	− 0.34	0.09
*SLC26A4*	0.12	0.53	− 0.072	0.71	− 0.34	0.068
*CDH23*	− 0.4	0.064	− 0.24	0.28	− 0.32	0.15

cVEMP, cervical vestibular-evoked myogenic potentials; oVEMP, ocular vestibular-evoked myogenic potentials; PTA, pure-tone audiometry.

Table 5 shows the correlation between age and vestibular function. A weak negative correlation was observed between age and each vestibular function test in controls (Rho = − 0.37 for caloric testing; Rho = − 0.33 for cVEMP and oVEMP). In patients with the *GJB2* variants, the negative correlation between cVEMP and oVEMP was increased compared with that in controls (cVEMP; Rho = − 0.66, *P* < 0.01; oVEMP: − 0.49, *P* = 0.01). Additionally, the negative correlation in caloric testing was stronger than that in controls in the *SLC26A4* variants cases (Rho = − 0.51, *P* < 0.01). In cases of *CDH23* variants, the negative correlation increased compared to the normal controls, with strong negative correlations observed between age and both cVEMP (Rho = − 0.83, *P* < 0.01) and oVEMP (Rho = − 0.88, *P* = 0.01).

**Table 5:** Correlation between age and vestibular function.

Age	Caloric testing		cVEMP		oVEMP	
	Rho	*P*-value	Rho	*P*-value	Rho	*P*-value
*GJB2*	− 0.13	0.54	− 0.66	0.0003	− 0.49	0.01
*SLC26A4*	− 0.51	0.004	− 0.39	0.03	− 0.39	0.03
*CDH23*	− 0.35	0.11	− 0.83	< 0.001	− 0.88	< 0.001
Control	− 0.37	< 0.001	− 0.33	0.03	− 0.33	0.003

cVEMP, cervical vestibular-evoked myogenic potentials; oVEMP, ocular vestibular-evoked myogenic potentials.

## Discussion

When comparing vestibular function between each gene, 69% of patients with *GJB2* variants, 73% of those with *SLC26A4* variants, and 54.5% of those with *CDH23* variants displayed at least one form of vestibular dysfunction in the semicircular canal, saccule, or utricle. Major genes responsible for HL have been suggested to also cause vestibular dysfunction.

The cochlea and vestibule are histologically adjacent and evolutionarily and embryologically similar. Our previous review showed that numerous genes expressed in the cochlea are also expressed in the peripheral vestibular organs^21^. Therefore, patients with variants in genes responsible for HL may exhibit a phenotype of vestibular dysfunction in addition to the HL phenotype. In a recent comprehensive study on the vestibular phenotype in patients with hereditary HL, decreased vestibular function in caloric tests, cVEMP, and oVEMP was observed in 42%, 57.8%, and 85% of patients, respectively^25^, indicating that vestibular dysfunction is common in patients with hereditary HL. However, few studies have specifically investigated vestibular function in patients with hereditary HL. To the best of our knowledge, this is the first study to compare vestibular function in patients with the major causative genes associated with HL.

In this study of vestibular function in patients with *GJB2* variants, nine out of 13 patients (69%) had a decreased or absent reaction to cVEMP, with a significantly higher frequency compared with that in patients with *SLC26A4* and *CDH23* variants. Both our study and other previous reports also showed that 60–80% of patients with *GJB2* variants exhibit pathological cVEMP results^26–28^. Most pathological cVEMP results in the present study and previous reports indicate that variants in *GJB2* could frequently induce saccular defects. A human temporal bone study of compound heterozygous 35*del*G variants also showed cochlea saccular degeneration^29^. A previous study in mice showed that the survival of vestibular hair cells was observed in the utricle and ampulla of Cx30-/- mice, but Cx30 was required for the survival of saccular hair cells^30^. Because Cx26 and Cx30 are co-localized in the vestibular organ^31^, it has been suggested that they are co-assembled from Cx26 and Cx30; thus, Cx26 may not be required for the survival of the utricle and ampullae in a similar manner. In the present comprehensive study of vestibular function in patients with *GJB2* variants, all patients exhibited normal caloric responses, and 11 out of 13 patients (85%) showed normal oVEMP reactions. Most normal reactions in the caloric test and oVEMP indicated that the semicircular canal and utricular functions were intact. Although no previous reports have focused on utricular function in patients with *GJB2* variants, intact semicircular canal and utricular functions were confirmed in our clinical evaluation, consistent with findings from a mouse model and temporal bone study. Regarding the saccular hypofunction in *GJB2* variants, we had previously hypothesized that because a sacculus does not have dark cells, unlike the utricle and ampullae of the semicircular canal, the saccule is not able to maintain the endolymph, and saccular endolymph originates from the cochlea by longitudinal flow or diffusion; thus, the change in endolymph in the cochlea caused by *GJB2* variants may directly influence the saccular endolymph and induce degeneration and hypofunction of the saccule^26^.

Although no significant difference was observed in the frequency of semicircular canal dysfunction in patients with *CDH23* (27%) variants, 47% of patients with *SLC26A4* variants had semicircular canal dysfunction, which is more frequently observed in patients with other gene variants. In previous reports, the frequency of semicircular canal hypofunction in patients with EVA has varied from 33 to 87%^32–35^. However, patients with EVA do not always have *SLC26A4* variants. A previous study on caloric testing in patients with biallelic *SLC26A4* variants in China showed that 51.6% exhibited unilateral or bilateral vestibulopathies^36^. This result is consistent with that of the present study. Moreover, a previous report from Eastern Asia showed that 75% of patients with EVA experienced semicircular canal hypofunction^33^. In contrast, approximately 30% of patients with EVA had semicircular canal dysfunction in previous reports on Caucasian populations^34,35^. Because a higher prevalence of *SLC26A4* variants in patients with EVA has been reported in East Asians (80–98%) than in Caucasoid populations (20–40%)^17^, the higher frequency of semicircular canal dysfunction in EVA reports from East Asians than in Caucasoid populations may be due to *SLC26A4* variants.

In this study, the corrected amplitude of cVEMP in patients with *SLC26A4* variants was significantly higher than that in controls, with six ears of the three patients showing an extremely high amplitude (over 30 μV in oVEMP). Previous reports have also shown lower thresholds and higher cVEMP and oVEMP amplitudes in EVA cases^37,38^. The EVA is considered to function as a “third window” in which air-conducted sounds are shunted from the cochlea to the vestibule, creating air-bone gaps of hearing at lower frequencies^39^. Additionally, because air or bone vibration could be shunted by a third window, such as EVA, greater stimulation is thought to be transmitted to the sacculus and utricle in patients with EVA than in normal patients, resulting in lower thresholds and higher amplitudes of cVEMP and oVEMP^37,38,40^. Previous reports have mainly focused on EVA, and there are no reports involving cases with *SLC26A4* variants. The higher amplitudes of cVEMP and oVEMP observed in our results are likely attributed to the effect of the third window associated with EVA. However, this effect was not present in all cases, as lower amplitudes of cVEMP and oVEMP were observed in 20% and 40% of cases, respectively.

The *SLC26A4* gene encodes pendrin, an anion exchange protein^41^. In the inner ear, pendrin is predominantly expressed in epithelial cells of the cochlear duct, vestibular organs, and endolymphatic sac^42^. Although the pathological mechanism of *SLC26A4* variants remains elusive, studies on *SLC26A4* knock-out mice have shown that the failure to express pendrin is related to the onset of enlargement of the endolymphatic space at the embryonic stage, and pendrin expression is required for embryonic- development of the inner ear^43,44^. Enlargement of the endolymphatic duct during embryonic development causes endolymphatic hydrops (ELH).

Additionally, the loss of pendrin function also causes acidification, inhibition of calcium reabsorption, and loss of endocochlear potential in the endolymphatic space^41–43,45^. Enlargement of the endolymphatic duct and changes in the endolymphatic environment are believed to lead to the extension of epithelial cells and impair cell-to-cell transmission, ultimately causing degeneration of the entire inner ear^43,45^. However, *SLC26A4* knockout mice are completely deaf and show severe vestibular dysfunction, along with severe degeneration of hair cells in both the organ of Corti and the vestibule. These phenotypes may not always be considerably less severe in humans. Recently, the visualization of ELH has become possible using enhanced 3T magnetic resonance imaging (MRI)^46^. Our previous enhanced 3T-MRI study showed that all five patients with EVA and biallelic *SLC26A4* variants exhibited significant ELH^47^. This finding suggests that human variants in *SLC26A4* induce a reduction in embryonic expression or dysfunction of pendrin and may also be associated with the development of ELH and environmental changes in the cochlear and vestibular end-organs.

These findings indicate that vestibular symptoms and dysfunction caused by *SLC26A4* gene variants are influenced by multiple factors, including the effect of the third window due to EVA, changes in inner ear composition associated with ELH, and dysfunction or degeneration of vestibular endo-organs due to *SLC26A4* variants. It is difficult to understand why semicircular canal hypofunction occurs more frequently than otolithic hypofunction. One possible reason for this is that because the amplitude of VEMPs was originally high in these patients, even if the amplitude decreases, it may still be interpreted as a normal reaction.

Variants in *CDH23* cause both DFNB12 and USH1D^8,9^. USH1 is characterized by profound congenital HL, vestibular dysfunction, and retinitis pigmentosa^48^. Focusing on the vestibular function of USH1, Maliulo et al. showed that among the patients with USH1 studied, two out of three exhibited pathological caloric tests, three out of four showed absent or abnormal cVEMP results, and all four displayed absent oVEMP results^49^. Astuto et al. reported that all 19 patients with USH1D who underwent caloric or rotary chair tests showed late age at ambulation and no vestibular reflex^12^. Penning et al. reported abnormal ambulation and vestibular function in patients with USH1D^50^. These findings represent a characteristic feature of vestibular dysfunction in USD1D. In previous reports, the patients with DFNB12 had normal vestibular function^12,48,50^. However, these studies involved a small number of patients, and detailed vestibular function in DFNB12 has not been extensively explored. In our study, abnormal results were observed in 21%, 18%, and 36% of cases for caloric testing, cVEMP, and oVEMP, respectively. In particular, the MSPV of caloric testing and oVEMP amplitudes were significantly lower than those of normal controls. These findings suggest the presence of some degree of vestibular dysfunction in patients with DFNB12. However, only one patient showed no response to oVEMP, and none showed areflexia in caloric testing or lack of reaction in cVEMP. Mild vestibular dysfunction is hypothesized to occur in patients with DFNB12. Null variants of *CDH23* in mice studies are associated with USH1D, characterized by the degeneration of stereocilia in hair cells of the cochlear and vestibular end-organs, resulting in profound congenital hearing loss and vestibular dysfunction^51–53^. In contrast, *CDH23* missense variants in mice, such as *salsa*^54^, *jera*^55^, and *erlong*^56^, serve as models of DFNB12, presenting with progressive HL and normal behaviors, with preserved vestibular tip links in *salsa* and *jera* mice. The intact vestibular hair cell observed in DFNB12 mice differs from that in our human vestibular function study, suggesting the possibility of mild vestibular dysfunction. Accurate vestibular function was not directly measured in any of the missense model mice, raising the possibility that the vestibular system may experience minor dysfunction even in the absence of histological disorders.

Regarding vestibular symptoms, 73% (11/15) of patients with *SLC26A4* variants complained of vestibular symptoms, whereas less than 10% of patients with *GJB2* (1/13) and *CDH23* variants (1/11) complained of vestibular symptoms. In a study on the vestibular symptoms of 627 patients with hereditary hearing loss, 22.8% had these symptoms; moreover, the symptoms were common among patients with *COCH* and *SLC26A4* variants^25^. Our previous large cohort study showed that only three of 75 (4%) patients with *GJB2* variants and one out of 25 (4%) patients with DFNB12 complained of episodes of vestibular symptoms^7,15^. Previous reports on *SLC26A4* variants by Miyagawa and Sugiura showed that approximately 50% of the patients with *SLC26A4* variants complained of vestibular symptoms^17,57^, whereas a report by Jung showed that only 23% of the patients complained of vestibular symptoms^36^. Compared with previous reports, the present results regarding the frequency of vestibular symptoms are relatively higher in patients with *SLC26A4* variants. Although the exact reason for the difference in the frequencies of vestibular symptoms for *SLC26A4* variants remains unknown, the incidence of vestibular symptoms in patients with *SLC26A4* variants is higher than that in those with the other two genes. Additionally, there are few cases of clinical complaints of vestibular symptoms in patients with *GJB2* variants and DFNB12. One possible reason for the absence of vestibular symptoms in patients with *GJB2* variants and DFNB12 may be vestibular compensation caused by congenital or slowly progressive pathologies.

In this study, no clear correlation was identified between hearing levels and vestibular function for any gene. This lack of correlation may be attributed to the small sample size for each gene. However, regarding age and vestibular function, especially in the patients with *CDH23* variants, a notable trend indicated a decrease in the amplitude of cVEMP (Rho = − 0.83) and oVEMP (Rho = − 0.88) with increasing age, showing a stronger correlation than that observed in normal controls (Rho = − 0.33; cVEMP and oVEMP). Most patients with *CDH23* variants have a high frequency of progressive HL^15^. In the present cases of *CDH23* variants, all four patients aged below 20 years displayed normal vestibular function, whereas six out of seven (86%) patients aged over 20 years showed vestibular dysfunction in at least one vestibular test. In studies of age-related HL mouse models, which are thought to harbor *Cdh23* variants, mild decreases in hair cell density were observed in the vestibular end-organs^58,59^. Given that lower frequencies were not reflected in this study, no correlation was found with hearing levels. In contrast to hair cells of the cochlea, those of vestibular organs are tuned to very low frequencies^60^. Thus, if HL progresses to lower frequencies with age, vestibular receptors may be affected, as well as the progressive pathology of HL.

Regarding *GJB2* variants, cVEMP showed an increased correlation. HL in *GJB2* variants is known to be a less progressive gene^7^, and the reason for the increased correlation between age and cVEMP is unclear. Long-term exposure to potassium influx may result in gradual deterioration of saccular function.

Regarding *SLC26A4*, a moderate correlation was observed between age and caloric testing. Although it was not associated with hearing levels, the characteristic clinical features in patients with *SLC26A4* variants include hearing fluctuation and repeated vertigo. Repeated hearing fluctuation and vertigo with age may lead to a reduction in semicircular canal function, resembling Meniere's disease.

Previous reports have demonstrated the existence of genotype–phenotype correlations in HL for each gene^7,15,17^. Because most patients in our cohort had similar variants, such as c.235*del*C in *GJB2*, c.2168A > G (p.H723R) in *SLC26A4*, and c.719C > T (p.P240L) in *CDH23*, determining a correlation in vestibular dysfunction between genotypes is challenging. Further genotypes should be evaluated to clarify these correlations.

In this study, trends in the characteristics of vestibular function were demonstrated in patients with each genetic mutation. However, one limitation of this study is the relatively small sample size. Hence, further studies should be conducted with a greater number of patients in the future.

## Conclusions

The findings of this study suggest that the major genes responsible for HL can cause vestibular dysfunction, with each gene exhibiting unique characteristics in terms of the degree and location of vestibular dysfunction. These characteristics are summarized below:

1. Variants in *GJB2* are likely to result in saccule dysfunction; however, dizziness is rare. Even with saccular dysfunction, symptoms are less likely to occur because the central nervous system has compensated since infancy.
2. Variants in *SLC26A4* are likely to cause semicircular canal dysfunction, and the frequency of semicircular canal dysfunction tends to be higher than that in other genes. Additionally, the frequency of vestibular symptoms was higher, a characteristic of these gene variants. This may be attributed to the combined effects of genetic hypofunction, EVA, and ELH.
3. Unlike USH1D, DFNB12 caused by *CDH23* variants displays a lower frequency of vestibular symptom and dysfunction. However, mild vestibular dysfunction is possible, potentially worsening with age.

These findings will also facilitate the clinical application of genetic counseling for these patients and their families.

### Supplementary Information

Supplementary Table S1.

## Data Availability

The datasets generated and/or analyzed in the current study are available from the corresponding author upon reasonable request.
